# BODIPY dyads and triads: synthesis, optical, electrochemical and transistor properties

**DOI:** 10.1186/s13065-018-0430-5

**Published:** 2018-05-11

**Authors:** Sompit Wanwong, Piyachai Khomein, S. Thayumanavan

**Affiliations:** 1 Polymer for Energy, Environment and Technology Research Group, Division of Materials Technology, School of Energy, Environment and Materials, King Mongkut’s University of Technology Thonburi, 126 Pracha Uthit Rd., Bang Mod, Thung Khru, Bangkok, 10140 Thailand; 2 Nanotec-KMUTT Center of Excellence on Hybrid Nanomaterials for Alternative Energy, King Mongkut’s University of Technology Thonburi, 126 Pracha Uthit Rd., Bang Mod, Thung Khru, Bangkok, 10140 Thailand; 3 Department of Chemistry, University of Massachusetts Amherst, Amherst, 10300 USA

**Keywords:** Donor–acceptor, Donor–acceptor–donor, BODIPY, Organic semiconductor, Transistor property, Surface morphology

## Abstract

A series of D–A dyads and D–A–D triads molecular systems based on triphenylamine and 9-ethyl-carbarzole as donor (D) and BODIPY as acceptor (A) has been designed and synthesized. The optoelectronic properties including optical, electrochemical, and charge carrier mobility of these molecules have been investigated. We found that the D–A–D triads exhibited broader absorption, raising the HOMO energy levels and increase hole carrier mobilities. Analysis surface morphology revealed that BODIPY containing carbazole demonstrated smooth film and no macro phase aggregation was observed upon thermal annealing.

**Electronic supplementary material:**

The online version of this article (10.1186/s13065-018-0430-5) contains supplementary material, which is available to authorized users.

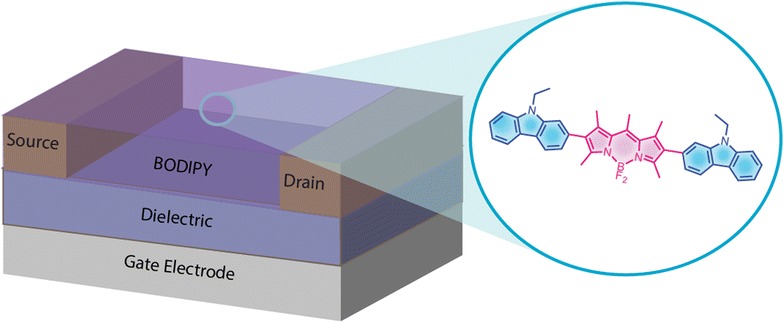

## Introduction

Organic semiconductors are crucial component in organic photovoltaics since they served as both light harvesting unit and charge transporting material that involved in energy conversion process. To effectively convert solar energy into electrical current, the organic semiconductors should have broad and intense absorption to harvest photon flux form the solar spectrum, proper HOMO and LUMO energy levels and sufficient charge carrier mobility to facilitate charge separation process [1, 2]. Typically, organic semiconductors consist of π-conjugated system which can be either small molecules or polymer based aromatic compounds. Polymer based semiconductors offer broader absorption, low cost deposition processing in small and large area [3–5]. However, they are polydisperse and tended to have batch-to-batch variation, higher molecular disorder and impurity from the end groups [6, 7]. In contrast, small π-conjugated molecules provide benefit on high purity with defined chemical structures, precise molecular weight and synthetically reproducible [6, 7]. These make small molecules gaining more attention to utilize in organic photovoltaics.

Recently, 4,4-difluoro-4-bora-3*a*,4*a*-diaza-*s*-indacen or boron dipyrromethene (BODIPY) has been explored for optoelectronic applications [8–10]. BODIPY is attractive heteroatom building block for organic semiconductor because it possesses excellent absorption properties with high molar absorptivity, high quantum yield and high photo-bleaching life time [11, 12]. The BODIPY core has three locations, the *meso*-position, the pyrrolic positions and the boron atom position, in which π-conjugation substituents can be attached [13, 14]. The effect of BODIPY structures on photophysical properties has been intensively explored [10, 13], while fundamental understanding on relationship between structures and charge transport property is less investigated. Generally, BODIPY based small molecules have been designed using symmetrical D–A–D and A–D–A triads [15, 16], where donor (D) is an electron rich functionality and acceptor (A) is a BODIPY unit. For instances, Krishnamoothy et al. [17] studied the effect of alkyl side chains at the *meso*-position of triphenylamine-BODIPY-triphenylamine triads on the charge transport properties. The hole mobilities of those BODIPY were found in the range of 10^−5^–10^−7^ cm^2^/V s. Recently, Thayumanavan et al. [18] developed the A–D–A structures where various donor moieties were incorporated at the *meso*-position of the BODIPY. They found that insertion of cyclopentadithiophene as the donor unit provided the highest electron mobility in the range of 10^−5^ cm^2^/V s. With the similar triad architecture, Facchetti et al. [19] reported that BODIPY-quaterthiophene-BODIPY can create crystalline fiber, resulting in high electron mobility of 10^−2^ cm^2^/V s. These examples suggest that charge transport characteristic of BODIPY can be either p-type (hole mobility) or n-type (electron mobility), depending on molecular architecture (D–A–D or A–D–A). Moreover, molecular packing of BODIPY is strongly influenced on charge mobility [17, 19].

**Fig. 1:**
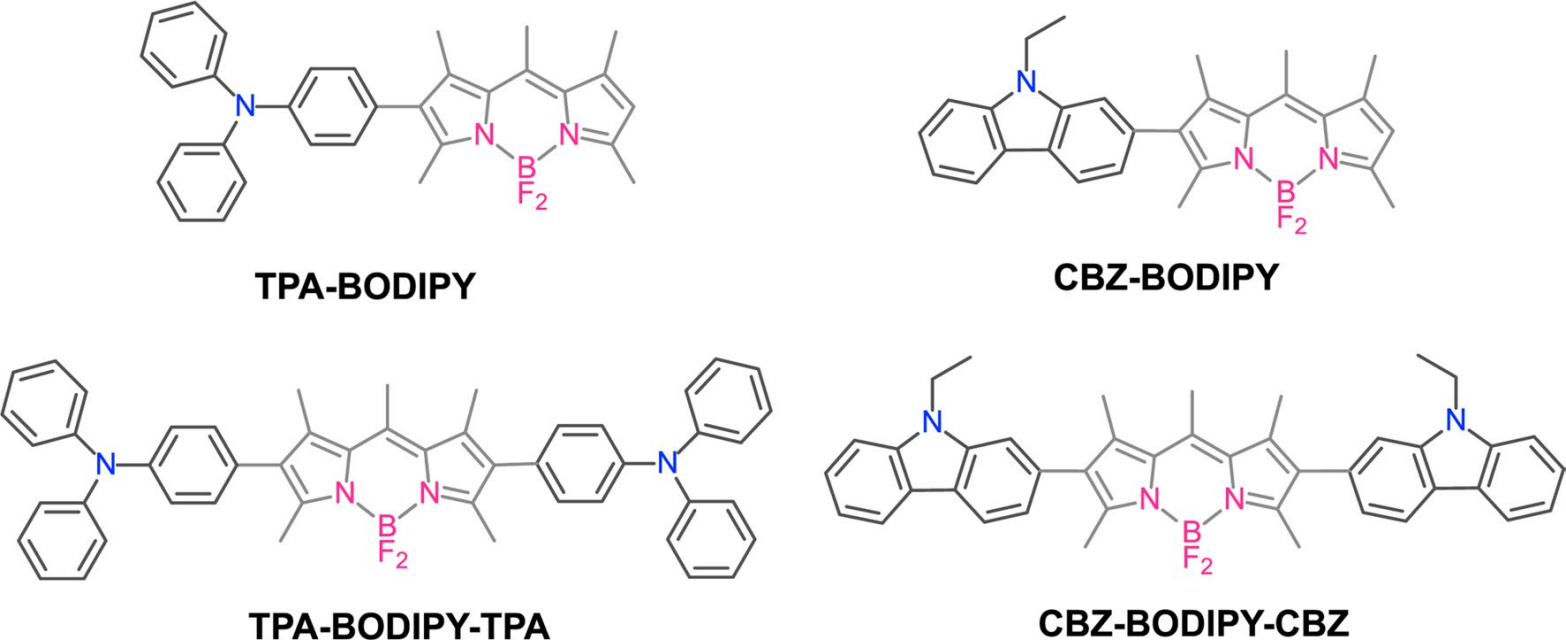
Chemical structures of **TPA-BODIPY**, **CBZ-BODIPY**, **TPA-BOIDPY-TPA** and **CBZ-BODIPY-CBZ**

Considering the variety of molecular systems, the relationship between D–A and D–A–D molecular structures on optical properties, charge mobility and surface morphology are of our interest. To broadening our understanding, we have designed to incorporate two different donor units on the BODIPY core. We chose triphenylamine (TPA) and carbazole (CBZ) as donor functionalities because they were easily oxidized into the radical cation and they have been largely employed in various optoelectronic devices [20, 21]. The donor units were attached at the 2- and 6-positions of the BODIPY acceptor to obtained dyad and triad structures. The substitution at the 2,6-position is expected to provide high degree of planarity and effective conjugation [13, 15]. Here, we described the optical, electrochemical of the BODIPYs, namely, **TPA-BODIPY**, **CBZ-BODIPY**, **TPA-BODIPY-TPA** and **CBZ-BODIPY-CBZ**, respectively (Fig. 1). The field-effect transistors were fabricated. The transistor properties and surface morphology of BODIPY derivatives have been investigated. The relationship between the resulting morphology and device performance is analyzed.

## Results and discussion

### Synthesis

**Scheme 1:**
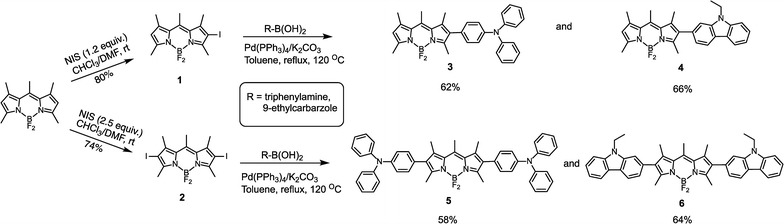
Synthetic route of BODIPY dyads and triads (**3**–**6**)

The target BODIPYs (**3**–**6**) were prepared in three steps as depicted in Scheme 1. First, BODIPY precursor was iodination using 1.2 equivalent of *N*-iodosuccinimide (NIS) to give 2-iodo-BODIPY (**1**) and 2.5 equivalent of NIS to give 2,6-diiodo-BODIPY (**2**) in good yield. The 2-iodo-BODIPY and the 2,6-diiodo-BODIPY were then incorporated with triphenylamine-4-boronic acid or 9-ethylcarbazole-3-boronic acid using Suzuki–Miyaura cross coupling reaction to yield the corresponding products (**3**–**6**), **TPA-BODIPY**, **CBZ-BODIPY**, **TPA-BODIPY-TPA** and **CBZ-BODIPY-CBZ**, respectively. The chemical structures were characterized using ^1^H-NMR, ^13^C-NMR and mass spectrometry to confirm their identity and purity (see Additional file 1).

### Absorption properties

**Fig. 2:**
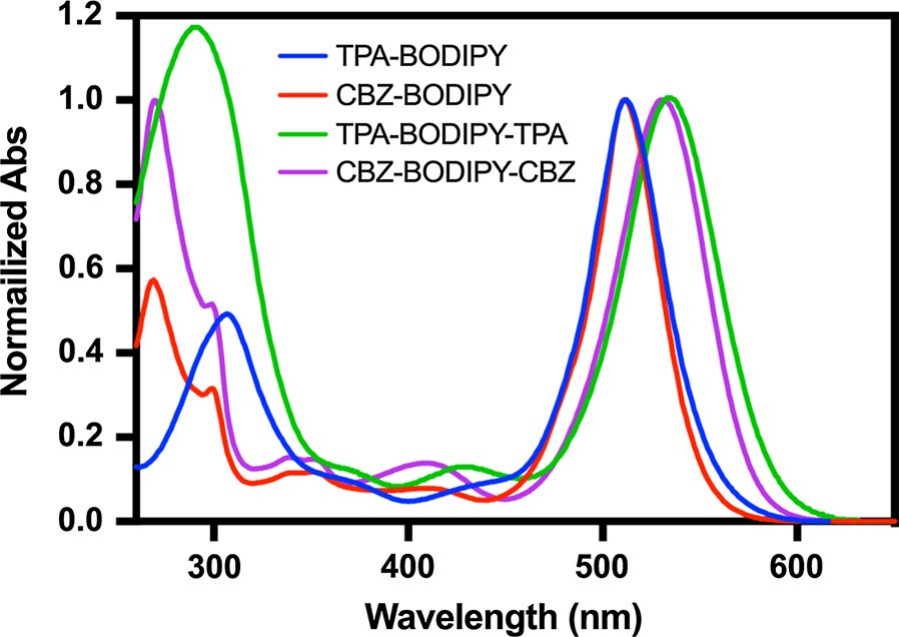
Absorption spectra of BODIPY derivatives (**3**–**6**)

The optical properties of BODIPY dyads and triads (concentration of 10^−5^ M) were examined in dichloromethane solution using UV–Vis spectrophotometry. The steady-state absorption spectra of D–A and D–A–D have shown in Fig. 2. The photophysical parameters including absorption maxima, molar extinction coefficient, absorption onset and optical spectra were determined, as shown in Table 1. The BODIPY dyads and triads exhibited two strong absorption bands at 269–307 and at 511–531 nm with the extinction coefficients in the range of 10^4^ M^−1^ cm^−1^. The first absorption band was attributed to the absorption of the donor moieties, triphenylamine and carbazole, whereas the second absorption band was attributed to the intramolecular charge transfer (ICT) from donor to the BODIPY unit. The absorption maxima of **TPA-BODIPY-TPA** and **CBZ-BOIDPY-CBZ** are red-shifted by 25 nm and 20 nm, comparing to those of **TPA-BODIPY** and **CBZ-BODIPY**, respectively. The longer absorption was due to the extended conjugation length of the BODIPY triads. The optical bandgaps were determined from the long wavelength absorption edge (λ_onset_) using the equation E_g_ = 1240/λ_onset_. As a result, the energy bandgap (E_g_) of **TPA-BODIPY**, **CBZ-BODIPY**, **TPA-BODIPY-TPA** and **CBZ-BODIPY-CBZ** were 2.05, 2.07, 1.98 and 2.00 eV, respectively. Thus, **TPA-BODIPY-TPA** exhibited the lowest energy bandgap. This is due to the strong electron donation ability of triphenylamine unit that enhance intramolecular charge transfer to the BODIPY acceptor unit [20, 22].

**Table 1:** Photophysical and electrochemical properties of BODIPY dyads and triads

Compounds	λ_max_ (nm)	ε_max_ (× 10^4^ M^−1^ cm^−1^)	λ_onset_ (nm)	E_g_, opt (eV)	E_ox_ (V)	HOMO^a^ (eV)	LUMO^b^ (eV)
**TPA-BODIPY**	307, 512	2.1, 4.3	605	2.05	+ 0.39	− 5.03	− 2.98
**CBZ-BODIPY**	269, 511	4.5, 7.9	599	2.07	+ 0.45	− 5.13	− 3.05
**TPA-BODIPY-TPA**	291, 537	4.3, 3.6	625	1.98	+ 0.23	− 4.92	− 2.94
**CBZ-BODIPY-CBZ**	270, 531	8.3, 8.3	619	2.00	+ 0.37	− 5.06	− 3.06

^a^HOMO energy is calculated from the equation, HOMO = − (E_ox_ + 4.8) [23]

^b^LUMO energy is calculated from the equation, E_g_, opt = LUMO − HOMO

### Electrochemical properties

**Fig. 3:**
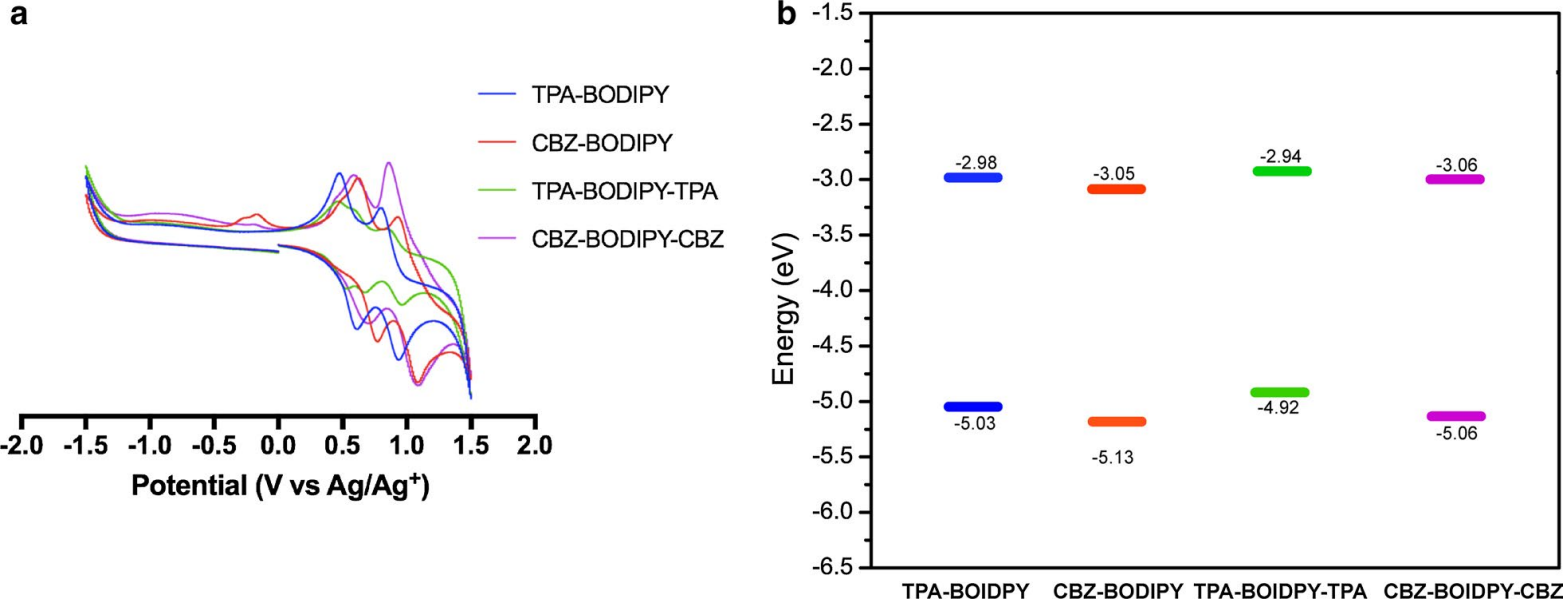
**a** Cyclic volttammograms of BODIPY dyads and triads in 0.1 M TBAPF_6_/CH_2_Cl_2_ measured against Ag/Ag + reference cell, ferrocene was added as the internal standard and **b** energy alignments of BODIPY dyads and triads

The electrochemical properties of BODIPY derivatives were analyzed using cyclic voltammetry. The solution of BODIPYs in anhydrous dichloromethane were prepared and the electrochemical characterization were performed using three-electrode system. The cyclic voltammogram and energy levels are illustrated in Fig. 3. The redox properties, the estimated HOMO and LUMO energy levels are summarized in Table 1. Cyclic voltammograms of all compounds exhibited only reversible oxidation wave. The oxidation potentials of **TPA-BODIPY**, **CBZ-BODIPY**, **TPA-BODIPY-TPA** and **CBZ-BODIPY-CBZ** were 0.39, 0.45, 0.23, and 0.37 V versus Ag/AgCl, respectively. These corresponded to the HOMO energy levels of − 5.03, − 5.10, − 4.92 and − 5.06 eV. Incorporation of triphenylamine units to the BODIPY core provided higher HOMO energy levels, suggesting that triphenylamine is stronger electron donor than carbazole. This is consistent to the longer λ_onset_ in the optical spectrum (Fig. 2). The LUMO energy levels were estimated using the optical bandgap.

### Charge transport properties

Charge carrier mobility of BODIPY dyads and triads were characterized in thin film using the bottom contact field effect transistor (FET). The charge carrier mobilities were calculated using the following equation [24, 25]:

$${\text{I}}_{\text{D}} = ({{\upmu{\text{CiW}}} \mathord{\left/ {\vphantom {{\upmu{\text{CiW}}} { 2 {\text{L}}}}} \right. \kern-0pt} { 2 {\text{L}}}})\left[ {\left( {{\text{V}}_{\text{GS}} {-}{\text{ V}}_{\text{T}} } \right)} \right]$$

where I_D_ is the current flowing between drain and source gates, μ is the mobility, C_i_ is the capacitance of the gate dielectric, W is the channel width, L is the channel length, V_GS_ is the gate-source voltage and V_T_ is the threshold voltage.

**Fig. 4:**
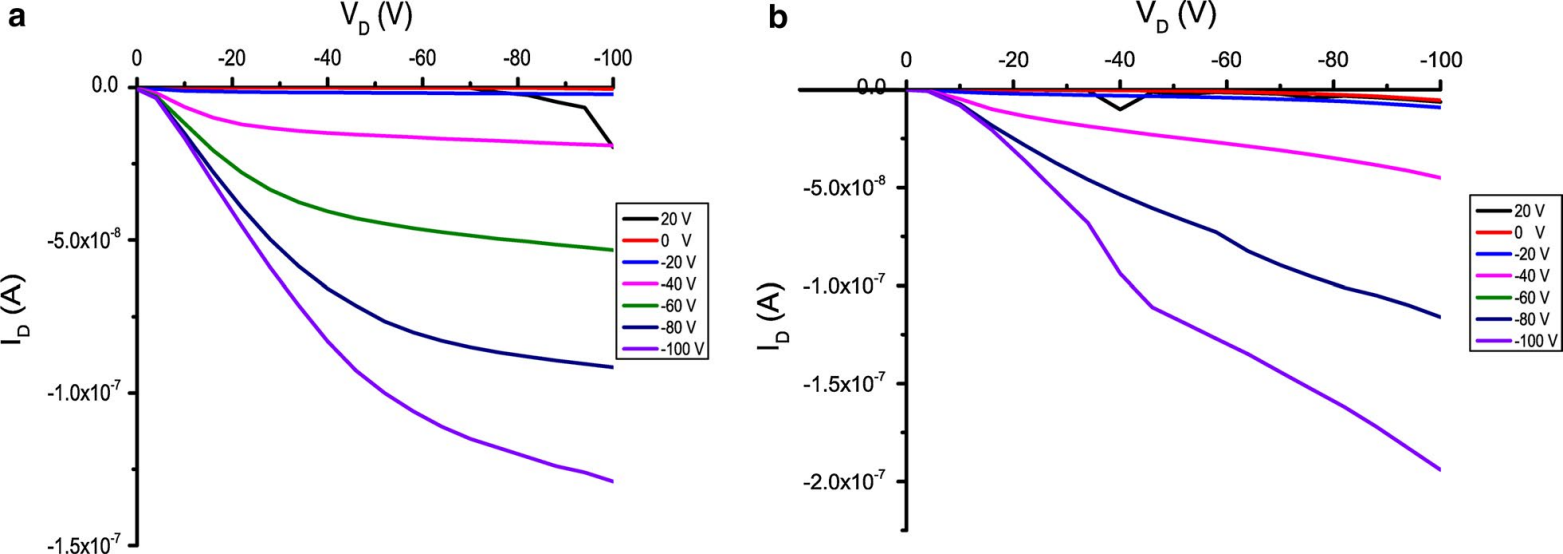
Output characteristics of **CBZ-BODIPY-CBZ a** before and **b** after annealing

Charge transport characteristic of BODIPY derivatives has been tested for both hole and electron mobility. The measurements were carried in a glovebox under argon atmosphere. The carrier mobility, threshold voltage and on/off current ratio are summarized in Table 2. We found that all compounds demonstrate only hole mobility, thus they behave as p-type semiconductors. For the D–A dyads system, the mobility of **TPA-BODIPY** and **CBZ-BODIPY** were found to be 9.27 × 10^-7^ and 5.29 × 10^−8^ cm^2^/V s, respectively. Increasing conjugation length of D–A–D triads provided higher mobility and increase of on/off current ratio. As seen in Table 2, **TPA-BODIPY-TPA** and **CBZ-BODIPY-CBZ** exhibited mobility of 1.66 × 10^−6^ and 7.86 × 10^−6^  cm^2^/V s, respectively. The increased hole mobility could be correlated to the higher HOMO energy levels of the D–A–D system that facilitate hole charge to travel to the Fermi level of the gold metals. After annealing samples at 80°C in a glovebox under argon atmosphere for 3 h, the mobilities of BODIPYs were improved by over an order of magnitude. As a result, **CBZ-BODIPY-CBZ** obtained the highest mobility of 2.95 × 10^−5^ cm^2^/V·s (Fig. 4b). This value is comparable to other reported BODIPY based small molecules that exhibit uniform film [17, 18].

**Table 2:** Organic thin-film transistor characteristics of BODIPY dyads and triads

Compounds	μ_h_ (cm^2^ V^−1^ s^−1^) before annealing	μ_h_ (cm^2^ V^−1^ s^−1^) after annealing	V_T_^a^ (V)	I_on_/I_off_^a^
**TPA-BODIPY**	9.27 × 10^−7^	1.55 × 10^−5^	23	7.3
**CBZ-BODIPY**	5.29 × 10^−8^	7.37 × 10^−7^	69	5.6
**TPA-BODIPY-TPA**	1.66 × 10^−6^	5.10 × 10^−8^	42	10^2^
**CBZ-BODIPY-CBZ**	7.86 × 10^−6^	2.95 × 10^−5^	52	10^2^

^a^Before annealing

**Fig. 5:**
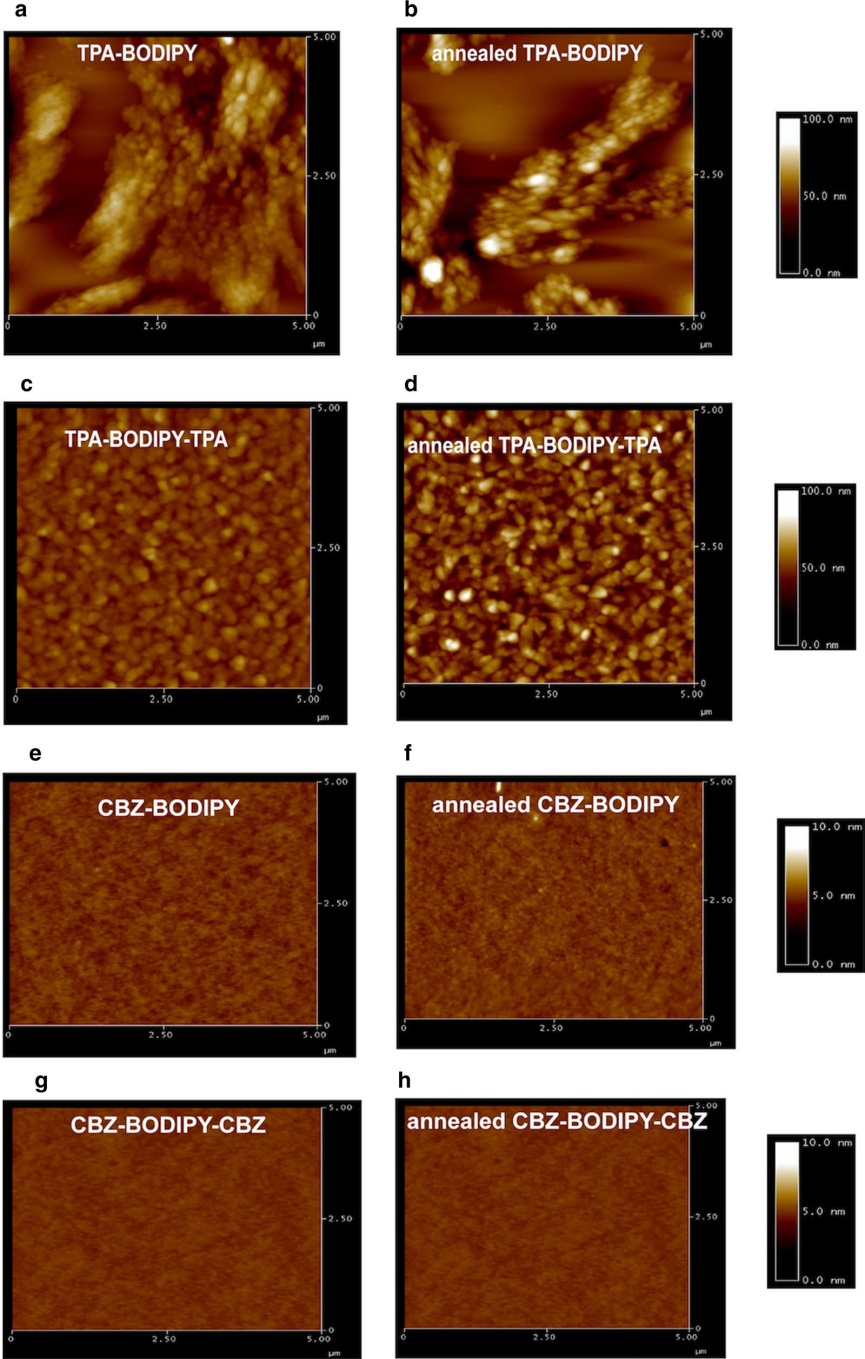
AFM topographic images

The difference in mobility are usually related to the difference of molecular packing in solid state, film morphology and the structural defects [26–28]. To gain more information, AFM has been conducted in a tapping mode. The **TPA-BODIPY**, **CBZ-BODIPY**, **TPA-BODIPY-TPA** and **CBZ-BODIPY-CBZ** showed completely different film textures (Fig. 5). **TPA-BODIPY** exhibited obvious large grain separation, while **CBZ-BODIPY** showed more homogeneous and smooth morphology with low root mean square of average height (RMS) (0.23 nm). The flatter film of **CBZ-BODIPY** could be attributed to more planarity of carbazole unit, resulting in reduced torsion between the donor and the BODIPY acceptor. For the triad architectures, **TPA-BODIPY-TPA** film showed terrace-like structure and increasing of surface roughness after annealing. This poor film morphology is consistent with deterioration of the hole mobility. On the contrary, **CBZ-BODIPY-CBZ** revealed smooth surface with RMS of 0.18 nm. Decreasing in surface roughness suggests that introduction of ethyl carbazole groups tend to create more uniform film, thus the higher charge carrier mobility could be related to well-organized surface.

## Conclusion

In summary, we have synthesized BODIPY dyads and triads using triphenylamine and carbazole as electron donating groups. The BODIPY triads demonstrated higher hole carrier mobilities, as compared to the BODIPY dyads. FET device containing **CBZ-BODIPY-CBZ** exhibited mobility as high as 2.95 × 10^−5^ cm^2^/V s. Although the BODIPY derivatives provided moderate performance, we hope that this work would benefit on rational design of the next BODIPY semiconductors to optimize their transistor properties.

## Experimental section

### Materials

All reagents were purchased from TCI chemicals, Aldrich and Fisher. Deuterated chloroform (CDCl_3_) was purchased from Cambridge Isotope Laboratories. Silica gel for column chromatography was purchased from Silicycle. All chemicals were used as received.

### Synthesis details

#### Synthesis of **2-iododo-BODIPY** (**1**)

[[(3,5-Dimethyl-1*H*-pyrrol-2-yl)(3,5-dimethyl-2*H*-pyrrol-2-ylidene)methyl]methane]-(difluoroborane) or BODIPY (0.5 g, 1.9 mmol) was dissolved in chloroform (20 mL) and the reaction mixture was degassed for 10 min. A solution of *N*-iodosuccinimide (NIS) (0.52 g, 2.3 mmol) in anhydrous DMF (4 mL) was slowly added to a solution mixture. The reaction mixture was stirred at room temperature for 24 h. After that, the crude mixture was extracted with ethyl acetate and water. The organic layers were dried over Na_2_SO_4_ and concentrated using a rotary evaporator. The crude mixture was then purified by column chromatography over silica with DCM/hexane as an eluent to give the product **1** as orange powder (0.6 g, 80%) ^1^H-NMR (500 MHz, CDCl_3_): δ (ppm), 6.10 (s, 1H, CH_PYR_), 2.59 (s, 3H, CH_3_), 2.54–2.52 (d, 6H, CH_3_), 2.40–2.38 (d, 6H, CH_3_) MALDI-TOF MS (*m/z*) calculated for C_14_H_16_BF_2_IN_2_ [M + Na], 388.0073, found 411.1373.

#### Synthesis of **2**,**6-diiodo-BODIPY** (**2**)

BODIPY precursor (0.35 g, 1.3 mmol) was dissolved in chloroform (20 mL) and the reaction mixture was degassed for 10 min. A solution of NIS (0.74 g, 3.3 mmol) in anhydrous DMF (5 mL) was slowly added to a solution mixture. The reaction mixture was stirred at room temperature for 2 days. After that, the crude mixture was extracted with ethyl acetate and water. The organic layers were dried over Na_2_SO_4_ and concentrated using a rotary evaporator. The crude mixture was then purified by column chromatography over silica with DCM/hexane as an eluent to give the product **2** as red powder (0.50 g, 74%). ^1^H-NMR (500 MHz, CDCl3): δ (ppm), 2.62 (s, 6H, CH_3_), 2.53 (s, 3H, CH_3_), 2.46 (d, 6H, CH_3_) MALDI-TOF MS (*m/z*) calculated for C_14_H_15_BF_2_I_2_N_2_, 513.9386, found 513.9382.

#### Synthesis of **TPA-BODIPY** (**3**)

2-Iodo-BODIPY (0.37 g, 0.94 mmol) and triphenylamine-4-boronic acid (0.35 g, 1.2 mmol) were dissolved in toluene (15 mL). The mixture was degassed for 10 min. Then, Pd(PPh3)_4_ (0.1 g, 0.09 mmol) and K_2_CO_3_ (2 M) were added. The reaction mixture was stirred under reflux under N_2_ atmosphere for 24 h. The reaction mixture was extracted with DCM and the organic layer was washed with water twice and dried over Na_2_SO_4_. The organic solvent was evaporated to dryness under reduced pressure. The residue was purified by column chromatography using DCM/hexane as the eluents to give **3** as an orange solid (0.3 g, 62%). ^1^H-NMR (500 MHz, CDCl_3_): δ (ppm), 7.29–7.26 (m, 4H, CH_AR_), 7.17–7.11 (m, 6H, CH_AR_), 7.07–7.03 (m, 4H, CH_AR_) 6.07 (s, 1H, CH_PYR_), 2.64 (s, 3H, CH_3_), 2.55–2.52 (d, 6H, CH_3_), 2.43 (s, 3H, CH_3_), 2.36 (s, 3H, CH_3_) ^13^C-NMR (125 MHz, CDCl_3_): δ (ppm), 153.49, 152.54, 147.65, 146.77, 141.37, 140.77, 137.03 133.22, 131.05, 129.29, 127.42, 1124.55, 123.05, 123.02, 121.24, 17.37, 16.58, 15.50, 14.12, 13.34 MALDI-TOF MS (*m/z*) calculated for C_32_H_30_BF_2_NO_3_ [M + Na], 528.2501, found 528.2523.

#### Synthesis of **CBZ-BODIPY** (**4**)

2-Iodo-BODIPY (0.13 g, 0.34 mmol) and 9-ethyl-carbazole-3-boronic acid (0.12 g, 0.51 mmol) were dissolve in toluene (15 mL) and degassed for 10 min. Then Pd(PPh_3_)_4_ (0.04 g, 0.003 mmol) and K_2_CO_3_ (2 M) were added. The reaction mixture was refluxed and stirred under N_2_ for 24 h. The reaction mixture was extracted with DCM and the organic layer was washed with water twice and dried over Na_2_SO_4_. The organic solvent was evaporated to dryness under reduced pressure. The residue was purified by column chromatography using DCM/hexane as the eluents to yield the product **4** as an orange powder (0.10, 66%). ^1^H-NMR (500 MHz, CDCl_3_): δ (ppm), 8.12 (d, *J *=* 7.5* Hz, 1H, CH_AR_), 7.94 (s, 1H, CH_AR_), 7.51–7.44 (m, 4H, CH_AR_), 7.31 (m, 1H, CH_AR_) 7.27 (m, 1H, CH_AR_), 6.09 (s, 1H, CH_PYR_), 4.44 (q, 2H, CH_2_) 2.65 (s, 3H, CH_3_), 2.58–2.56 (d, 6H, CH_3_), 2.44 (s, 3H, CH_3_), 2.37 (s, 3H, CH_3_), 1.48 (t, 3H, CH_3_) ^13^C-NMR (125 MHz, CDCl_3_): δ (ppm), 153.14, 141.33, 140.46, 140.21, 139.08, 137.46, 134.54, 132.19, 132.00, 128.01, 125.83, 124.03, 123.00, 122.70, 122.09. 121.04, 120.40, 118.93, 108.54, 37.59, 17.32, 16.72, 15.51, 14.41, 13.84, 13.37 MALDI-TOF MS (*m/z*) calculated for C_28_H_28_BF_2_N_3_ [M + Na], 455.2344; found 478.224.

#### Synthesis of **TPA-BODIPY-TPA** (**5**)

2,6-Diiodo-BODIPY (**2**) (0.20 g, 0.39 mmol) and triphenylamine-4-boronic acid (0.33 g, 1.1 mmol) were dissolved in toluene (15 mL). The solution was purged with nitrogen gas for 10 min. Then, Pd(PPh_3_)_4_ (0.07 g, 0.06 mmol) and K_2_CO_3_ (2 M) were added. The reaction mixture was stirred and refluxed for 48 h. After completion of the reaction, the mixture was cooled to room temperature, followed by extraction with DCM and water. The organic layers were dried over Na_2_SO_4_ and concentrated in vacuo. The crude mixture was then purified by column chromatography using DCM/hexane as the eluents to yield **5** (0.17 g, 58%) purple solid. ^1^H-NMR (500 MHz, CDCl3): δ (ppm), 7.30–7.27 (m, 8H, CH_AR_), 7.17–7.12 (m, 12H, CH_AR_) 7.09–7.03 (m, 8H, CHAR) 2.72 (s, 3H, CH_3_), 2.55 (s, 6H, CH_3_), 2.37 (s, 6H, CH_3_)^13^C-NMR (125 MHz, CDCl_3_): δ (ppm), 152.47, 147.67,146.79, 141.29, 136.74, 133.25, 132,25, 131.09, 129.30, 127.47, 124.57, 123.07, 122.03, 17.27, 15.61, 13.38 HRMS (MALDI-TOF MS) (*m/z*) calculated for C_50_H_43_BF_2_N_4_, 748.3549; found 748.3662.

#### Synthesis of **CBZ-BODIPY-CBZ** (**6**)

2,6-Diiodo-BODIPY (**2**) (0.20 g, 0.4 mmol) and 9-ethylcarbazole-3-boronic acid (0.26 g, 1 mmol) were dissolved in toluene (15 mL). The solution was degassed for 10 min. Next, Pd(PPh_3_)_4_ (0.07 g, 0.06 mmol) and K_2_CO_3_ (2 M) were added. After which, the reaction mixture was stirred under reflux for 48 h. The crude mixture was extracted with EtOAc and water. The organic layers were dried over Na_2_SO_4_ and concentrated under reduced pressure. The crude product was then purified by column chromatography using DCM/hexane as the eluents to provide **6** as maroon solid (0.16 g, 64%). ^1^H-NMR (500 MHz, CDCl_3_): δ (ppm), 8.16 (d, *J *=* 7.5* *Hz*, 1H, CH_AR_), 8.00 (s, 1H, CH_AR_), 7.55–7.47 (m, 6H, CH_AR_), 7.38 d, 2H, CH_AR_) 7.36–7.27 (m, 2H, CH_AR_), 4.47 (q, 4H, CH_2_) 2.79 (s, 3H, CH_3_), 2.61 (d, 6H, CH_3_), 2.45 (s, 6H, CH_3_), 1.54 (t, 3H, CH_3_) ^13^C-NMR (125 MHz, CDCl_3_): δ (ppm), 152.68, 141.21, 140.25, 139.11, 137.02 134.41, 132.21, 132.21, 128.11, 125.84, 124.22, 123.03, 122.77, 122.18. 121.45, 118.95, 108.56, 108.30, 37.65, 17.22, 15.63, 13.88, 13.41 HRMS (MALDI-TOF MS) (*m/z*) calculated for C_42_H_39_BF_2_N_4_ [M + Na], 671.3236; found. 671.4772.

### Instrumentations

^1^H-NMR spectra were recorded on 400 and 500 MHz Bruker NMR spectrometer and were reported in ppm using the solvents as the internal standard (CDCl_3_ at 7.26 ppm). ^13^C-NMR spectra were proton decoupled and recorded on a 100 MHz Bruker spectrometer using the carbon signal of the deuterated solvent as the internal standard. The exact mass measurements were recorded on Bruker Daltonics micrOTOF mass spectrometer. UV–vis absorption spectra were recorded on a Thermo Scientific UV-Genesys 10 s spectrophotometer. Electrochemical measurements were performed on a BASi Epsilon potentiostat. Charge carrier mobility was determined in field effect transistor (FET) mode using Agilent 4165C precision semiconductor parameter analyzer. Surface morphology of BODIPY films were characterized using a digital instrument dimension 3100 atomic force microscope (AFM). The cantilever specifications are described as follows: spring constant of 40 N/m, resonance frequency of 300 kHz, tip radius of 8 nm.

### FET fabrication

Bottom contacted field effect transistor (FET) devices were fabricated following our previous procedure [23]. Briefly, the BODIPYs (**3**–**6**), concentration of 10 mg/mL in dichlorobenzene, were deposited on the FET substrate using a spin coater. All mobility measurements were performed in a glove box under argon atmosphere at a temperature of 25 °C. Thermal annealing was done in glove box under argon atmosphere. Briefly, a hot plate was pre-heated to 80 °C. Then, FET devices were placed on the hot plate and they were heated directly at the constant temperature for 3 h. After that, the devices were removed from the hot plate and allowed to cool down to room temperature and the mobilities were measured again.

## Additional file

**Additional file 1.** NMR spectroscopic data, FET and AFM measurements of BODIPY dyads and triads.
