# Supplementary material for: BODIPY dyads and triads: synthesis, optical, electrochemical and transistor properties
Source: Chem Cent J. 2018 May 11;12:60. doi: 10.1186/s13065-018-0430-5 (PMC5945575; doi:10.1186/s13065-018-0430-5)
Supplement: Supplementary file 1 — Additional file 1. NMR spectroscopic data, FET and AFM measurements of BODIPY dyads and triads. [file 13065_2018_430_MOESM1_ESM.docx]

**Additional file 1**

**BODIPY Dyads and Triads: Synthesis, Optical, Electrochemical and Transistor Properties**

Sompit Wanwong ^1,2^*, Piyachai Khomein ^3^ and S. Thayumanavan ^3^

*^1^ Polymer for Energy, Environment and Technology Research Group, Division of Materials Technology, School of Energy, Environment and Materials, King Mongkut’s University of Technology Thonburi, 126 Pracha Uthit Rd., Bang Mod, Thung Khru, Bangkok 10140, Thailand.*

*^2^ Nanotec-KMUTT Center of Excellence on Hybrid Nanomaterials for Alternative Energy, King Mongkut’s University of Technology Thonburi, 126 Pracha Uthit Rd., Bang Mod, Thung Khru Bangkok 10140, Thailand.*

*^3^ Department of Chemistry, University of Massachusetts Amherst, 10300, USA.*

**Contents:**

1. ^1^H-NMR, ^13^C-NMR, and MS spectra of BODIPY dyads and triads 2-9
2. FET measurements 10
3. AFM roughness 11-12


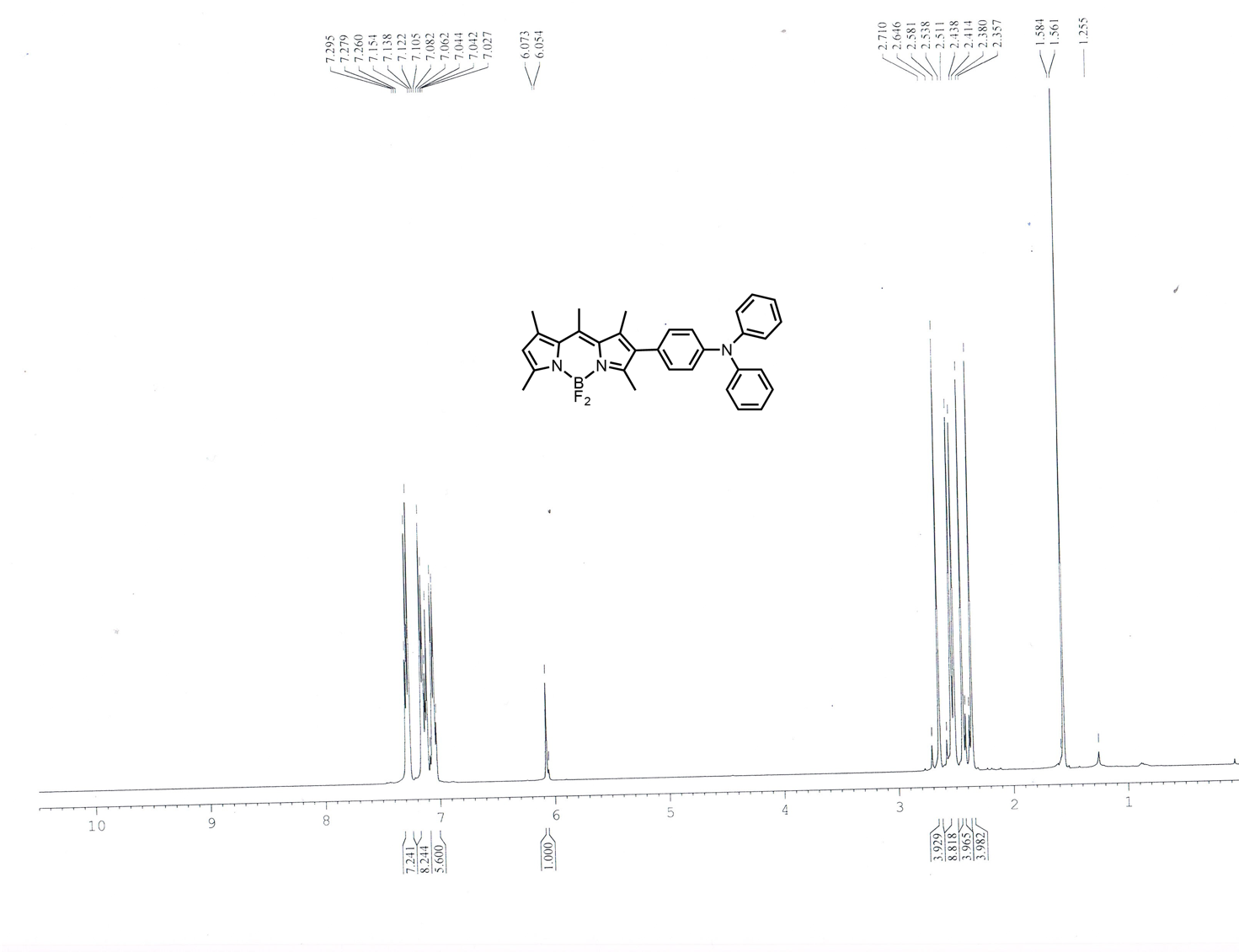


^1^H NMR of **TPA-BODIPY**


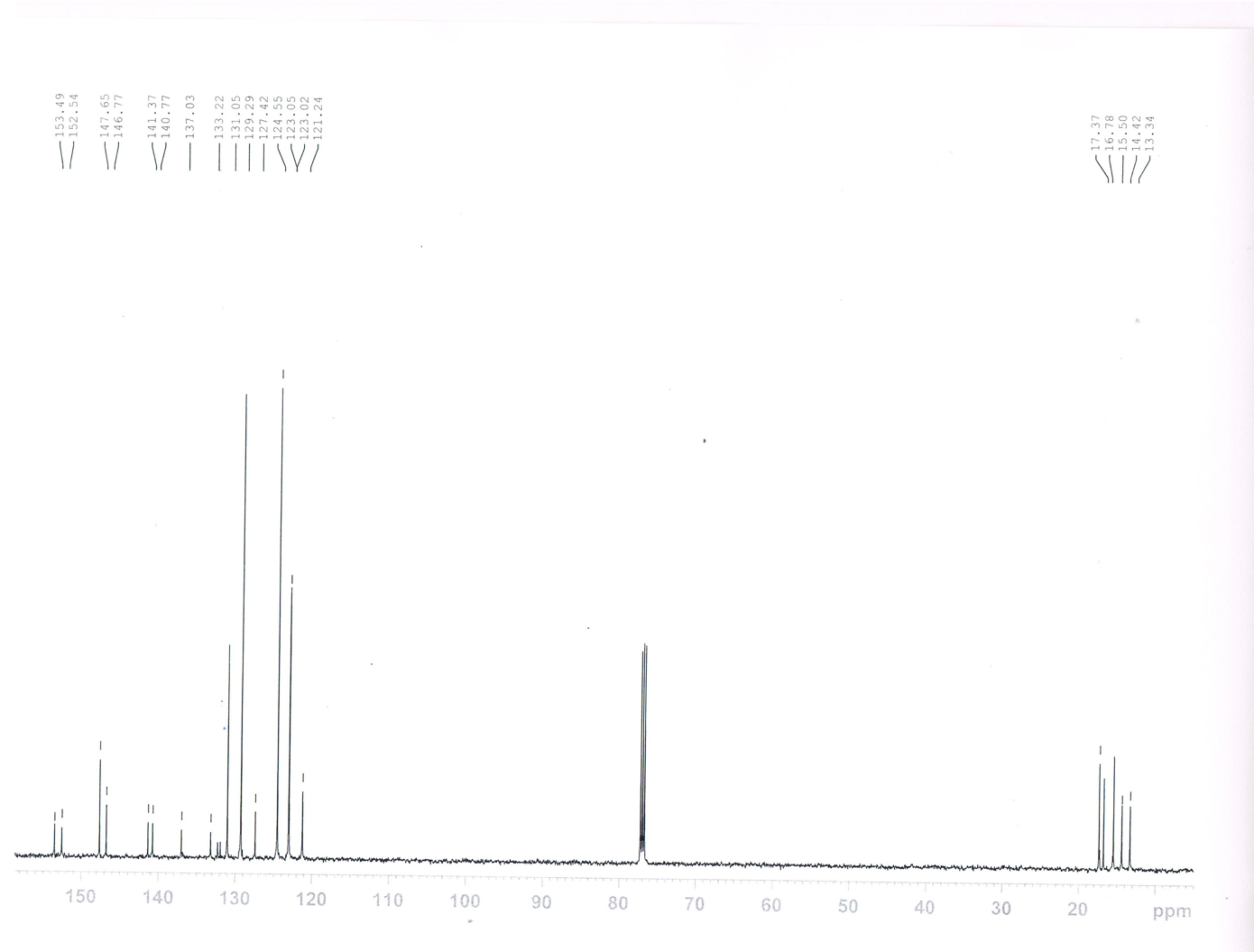


^13^C NMR of **TPA-BODIPY**


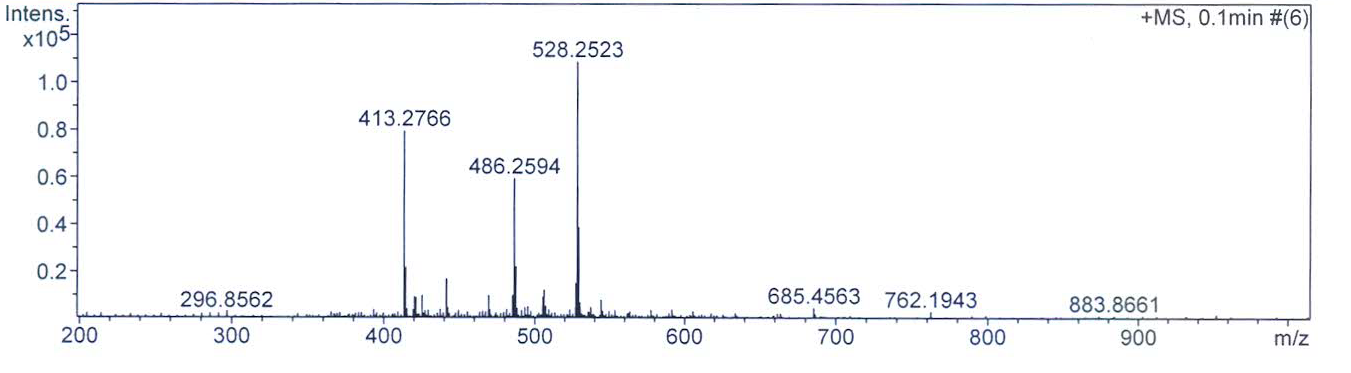


MS of **TPA-BODIPY**

*
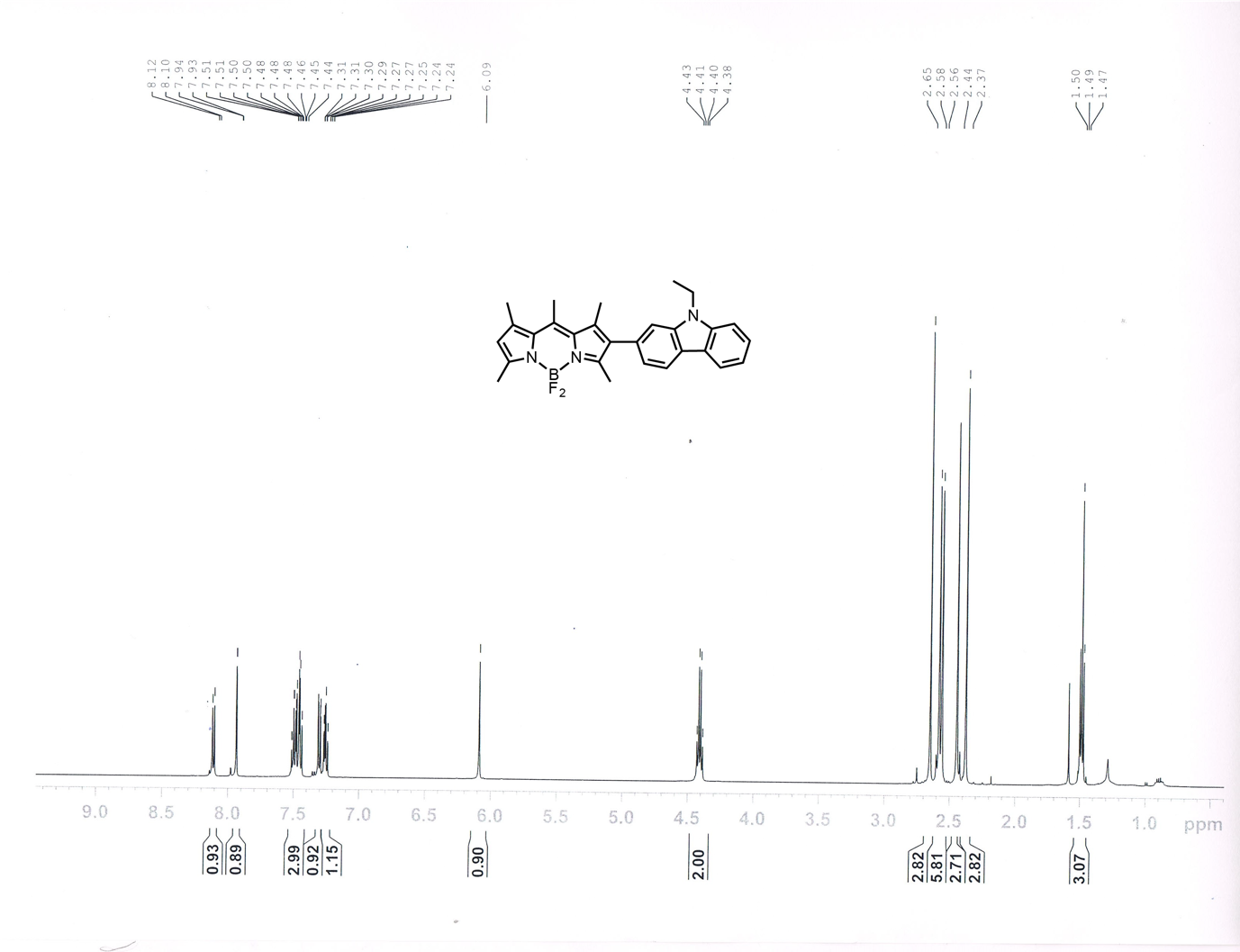
*

^1^H-NMR of **CBZ-BODIPY**


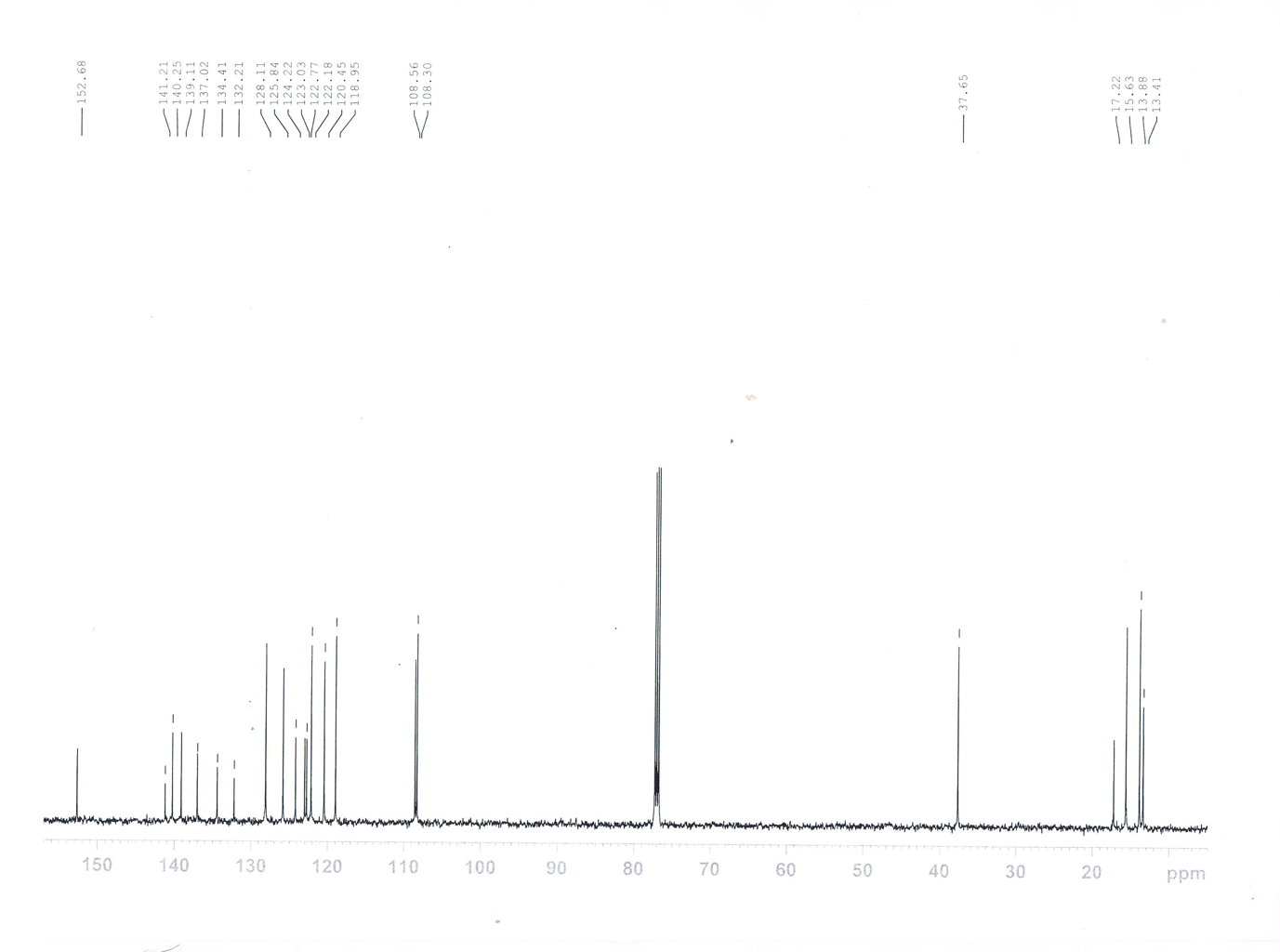


^13^C- NMR of **CBZ-BODIPY**


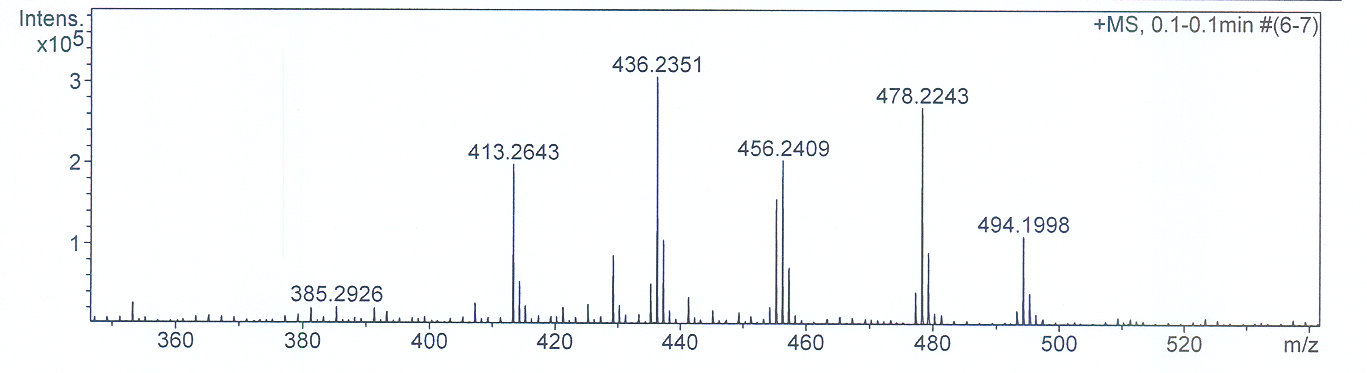


MS of **CBZ-BODIPY**


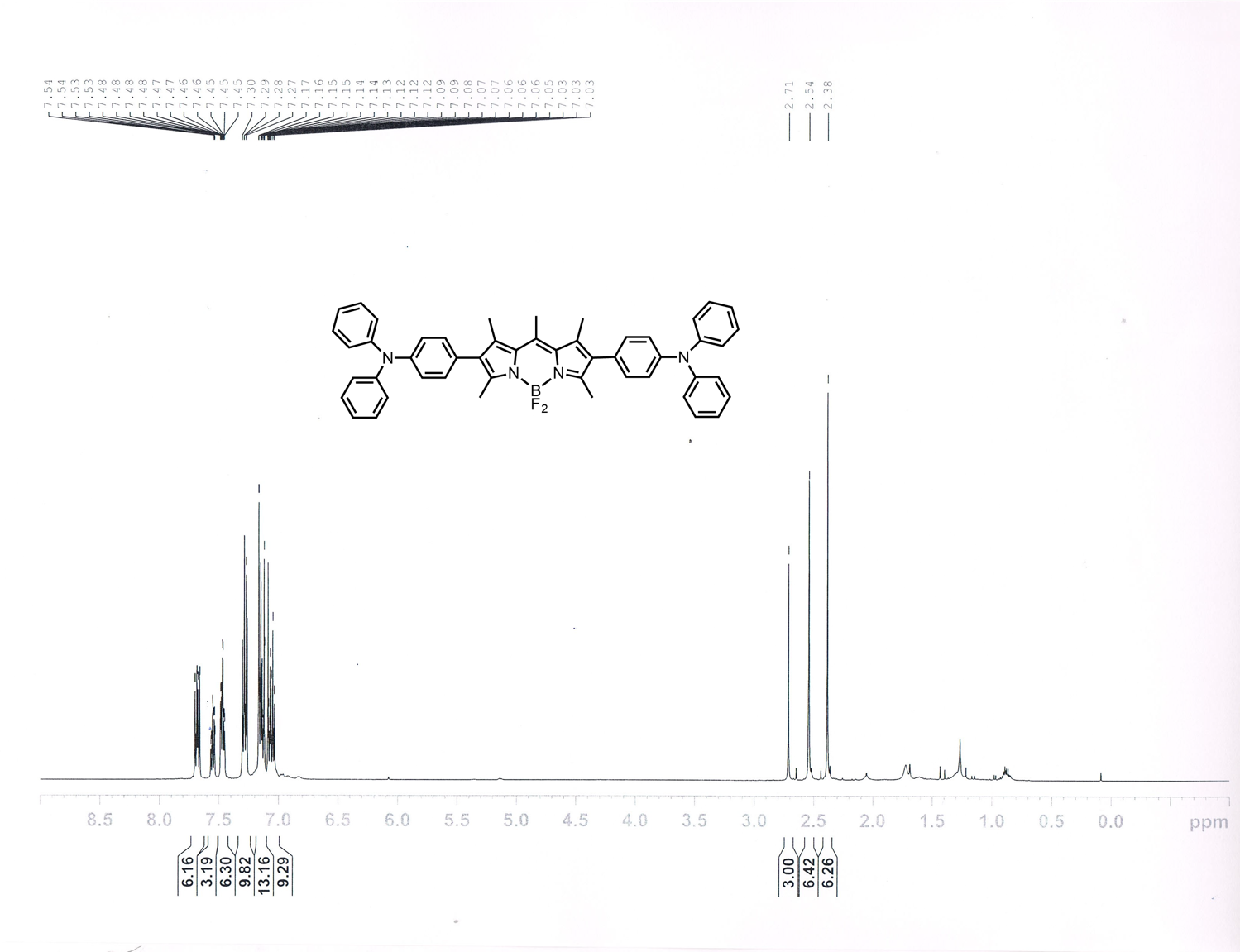


^1^H-NMR of **TPA-BODIPY-TPA**


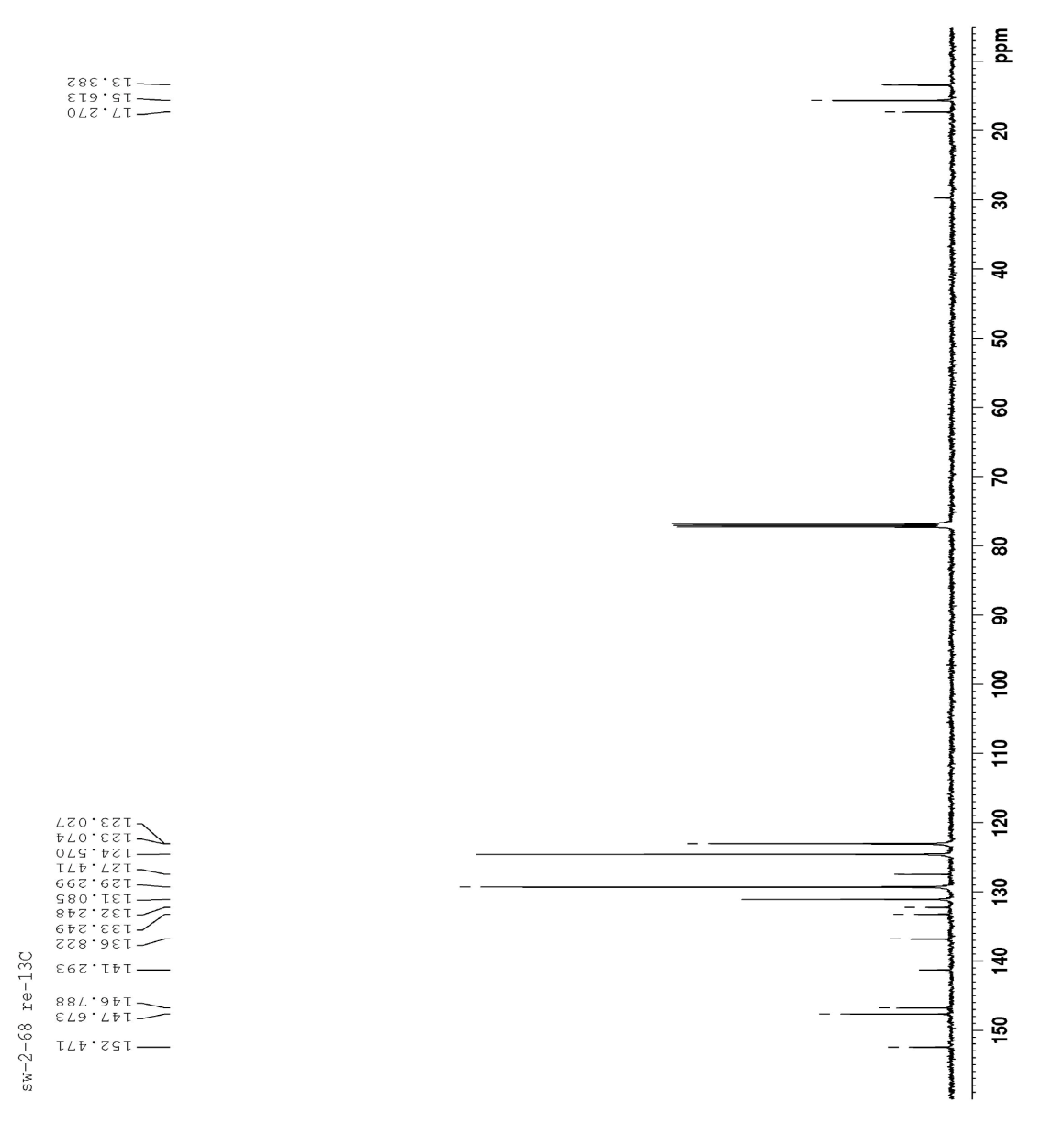


^13^C-NMR of **TPA-BODIPY-TPA**


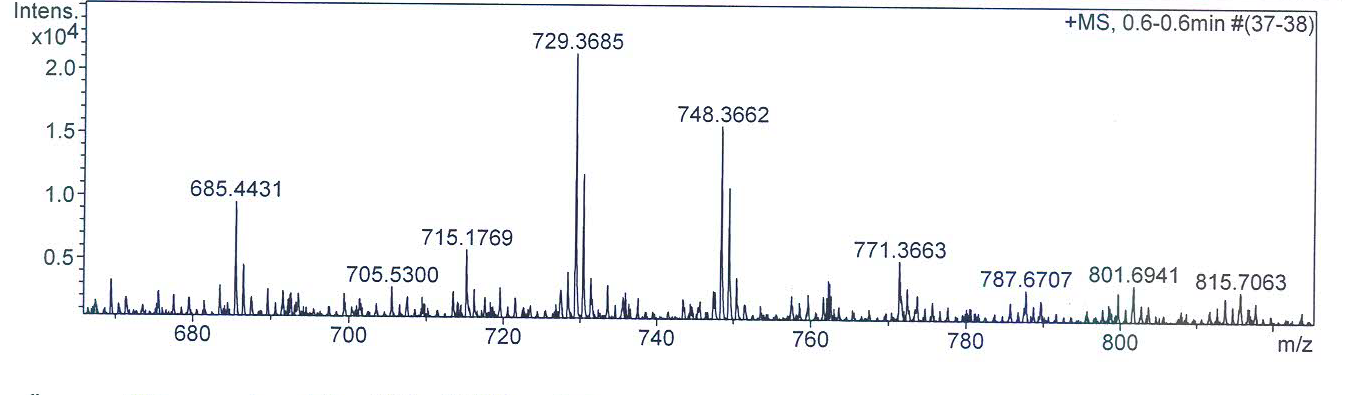


MS of **TPA-BODIPY-TPA**


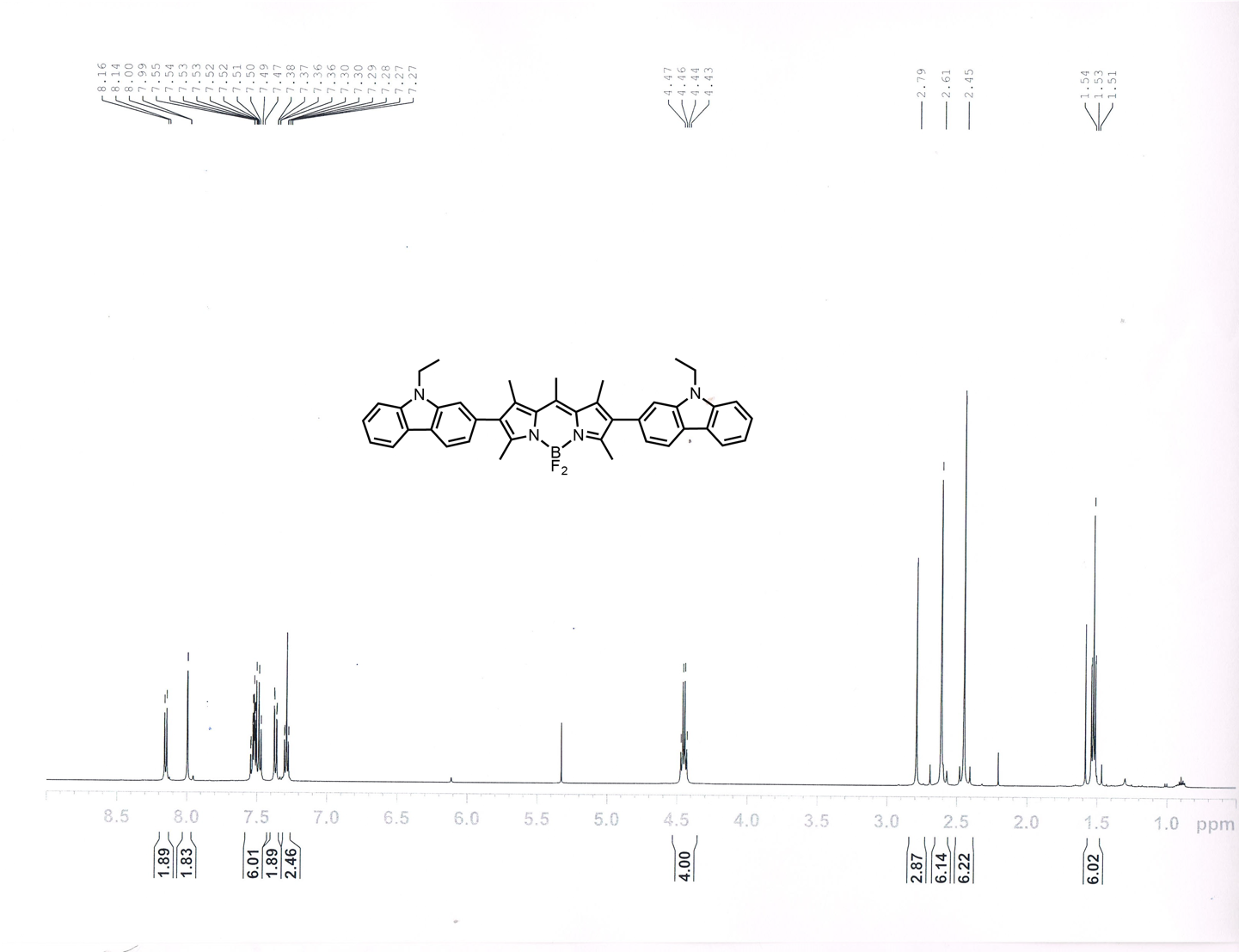


^1^H-NMR of **CBZ-BODIPY-CBZ**


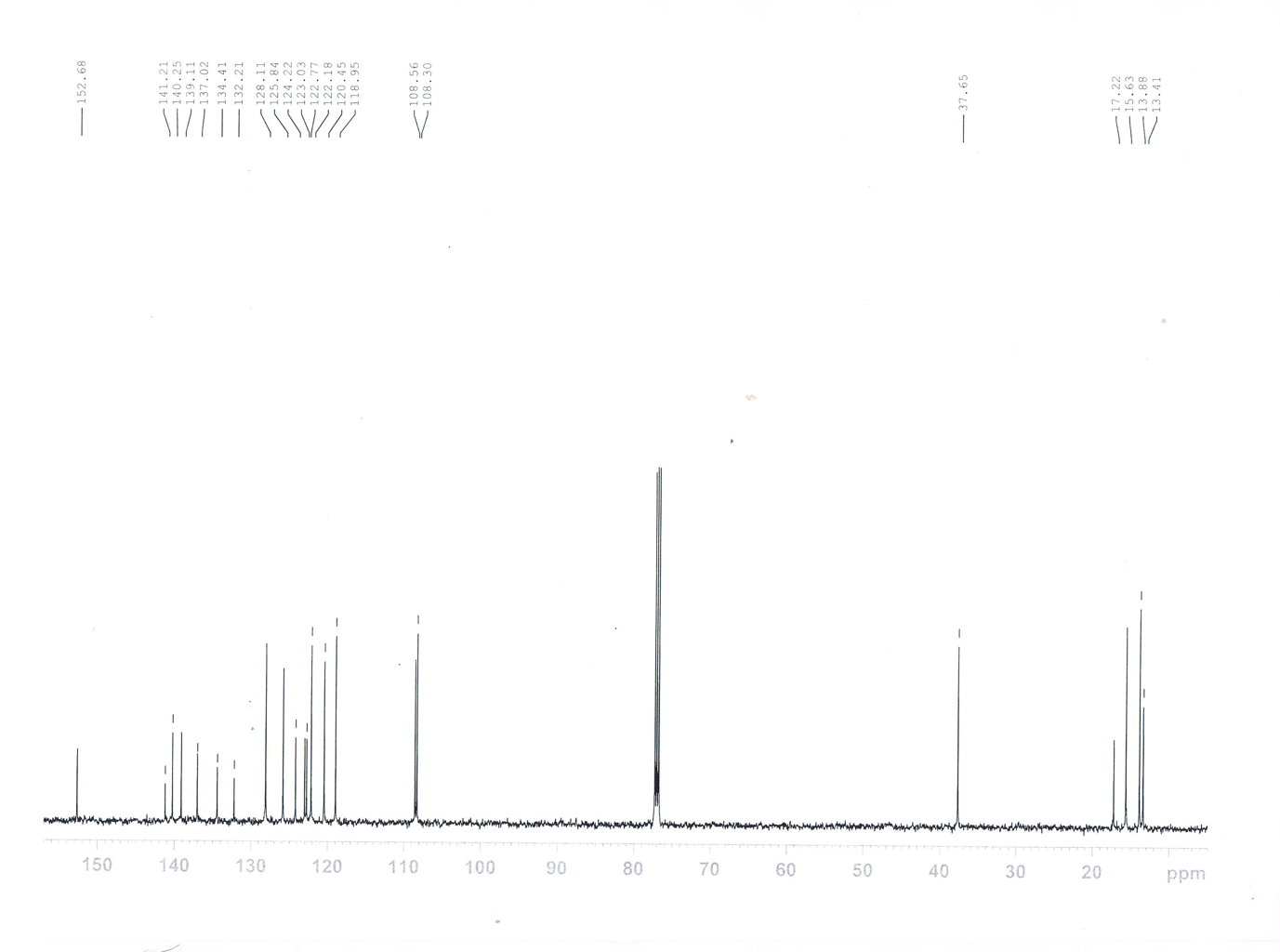


^13^C-NMR of **CBZ-BODIPY-CBZ**


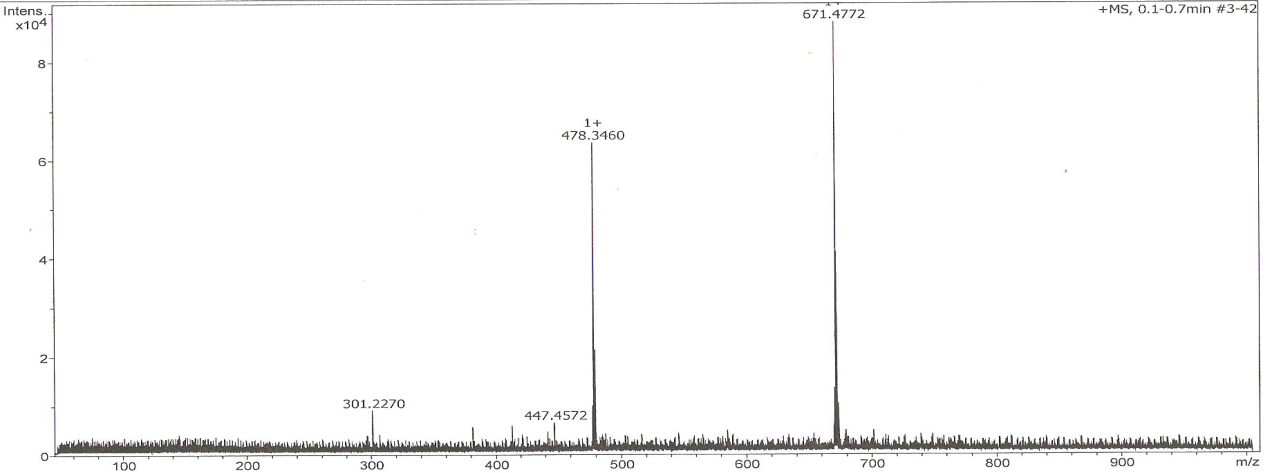


MS of **CBZ-BODIPY-CBZ**

**FET measurements**


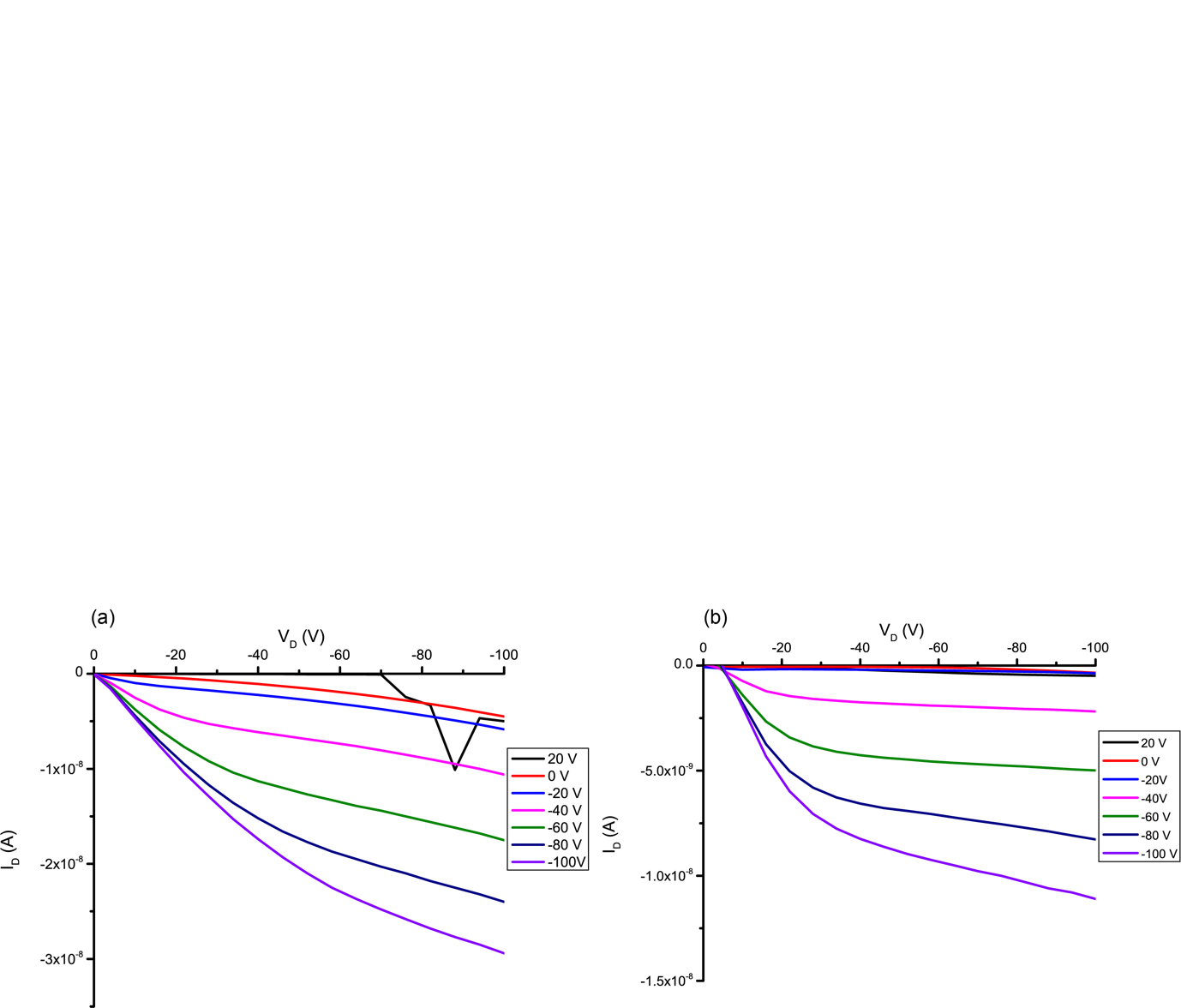


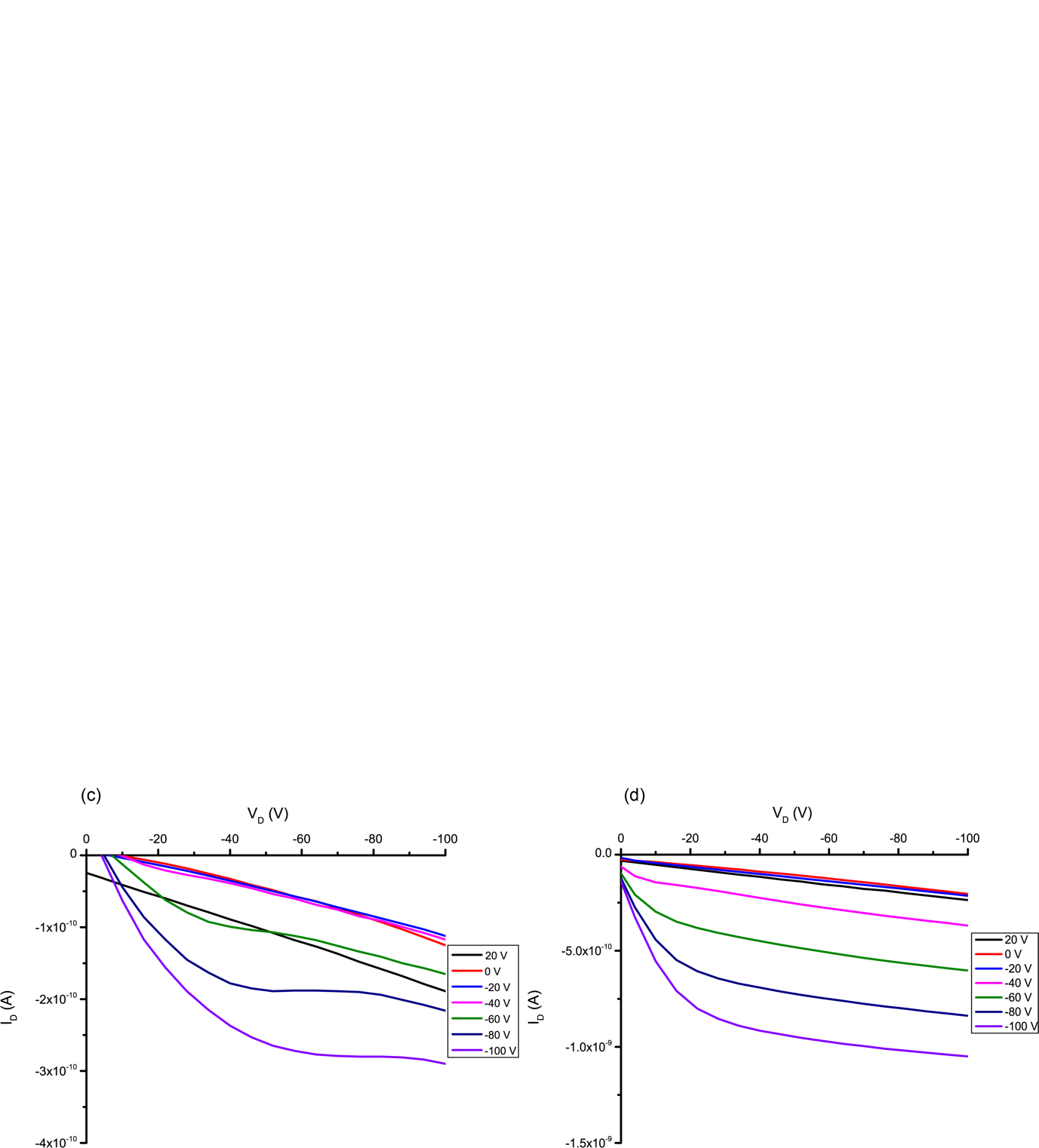


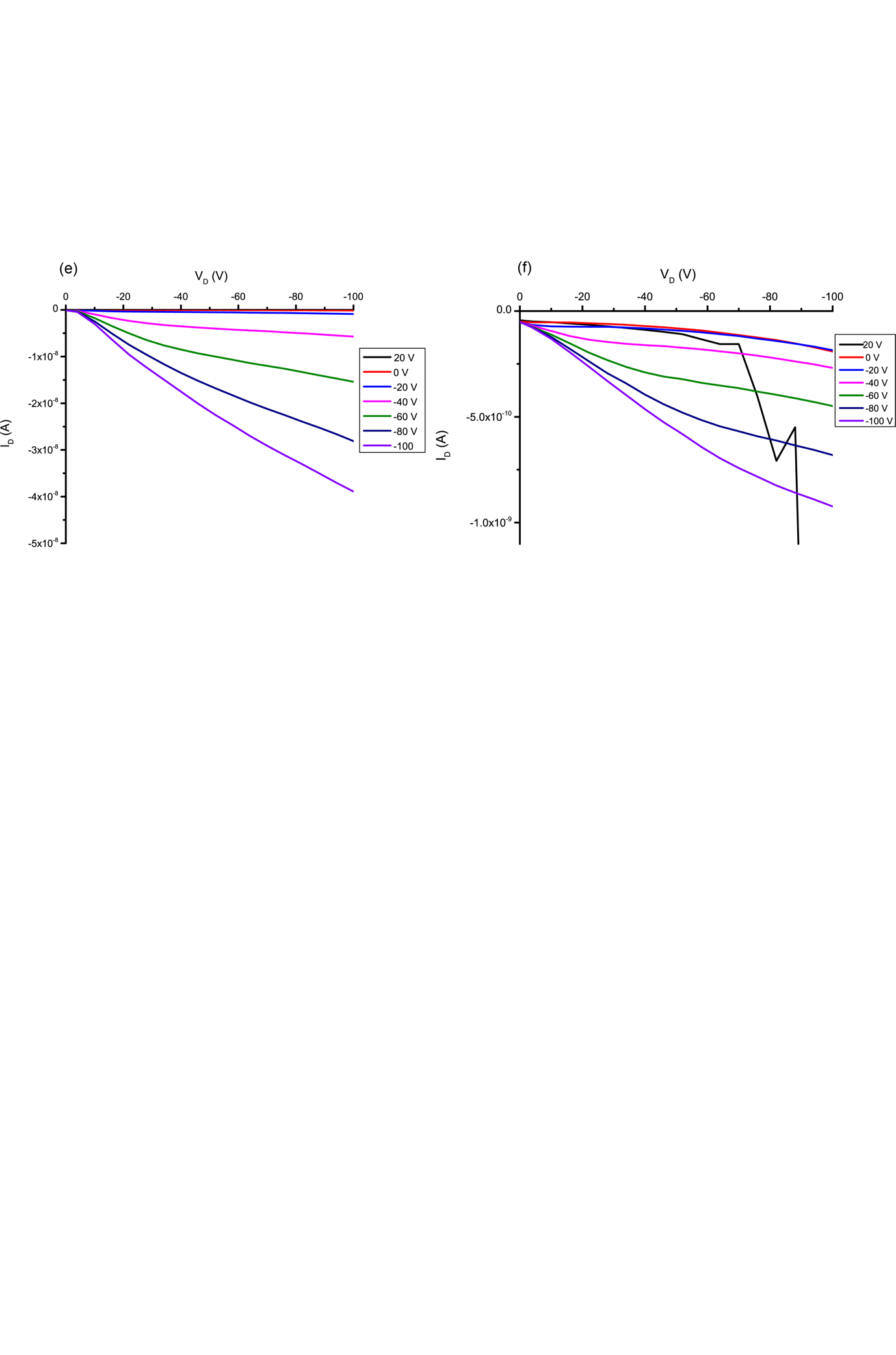


Output characteristics of (a) TPA-BODIPY, (b) annealed TPA-BODIPY, (c) CBZ-BODIPY, (d) annealed CBZ-BODIPY, (e) TPA-BODIPY-TPA, (f) annealed TPA-BODIPY-TPA

**AFM**


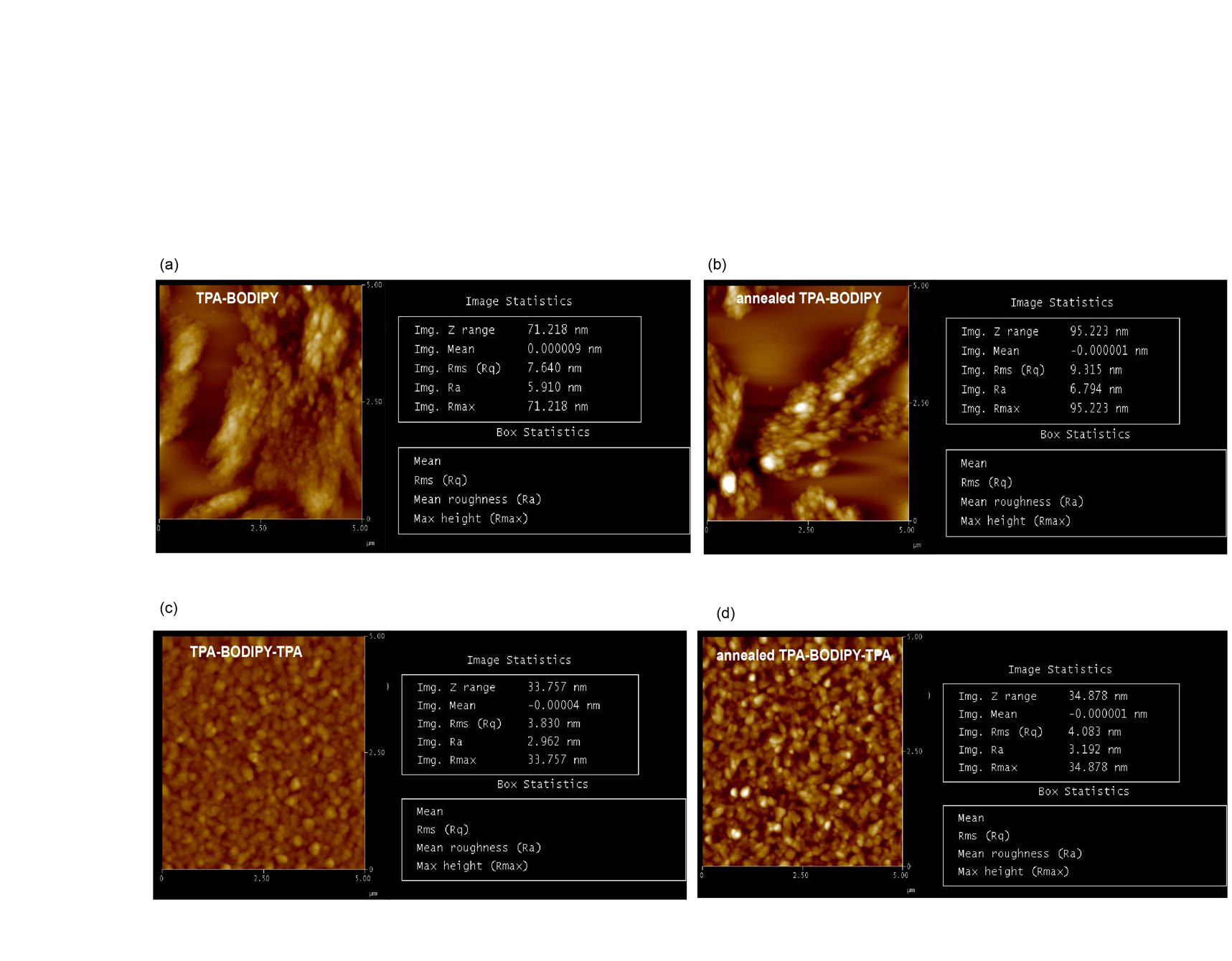


Surface roughness TPA-BODIPY


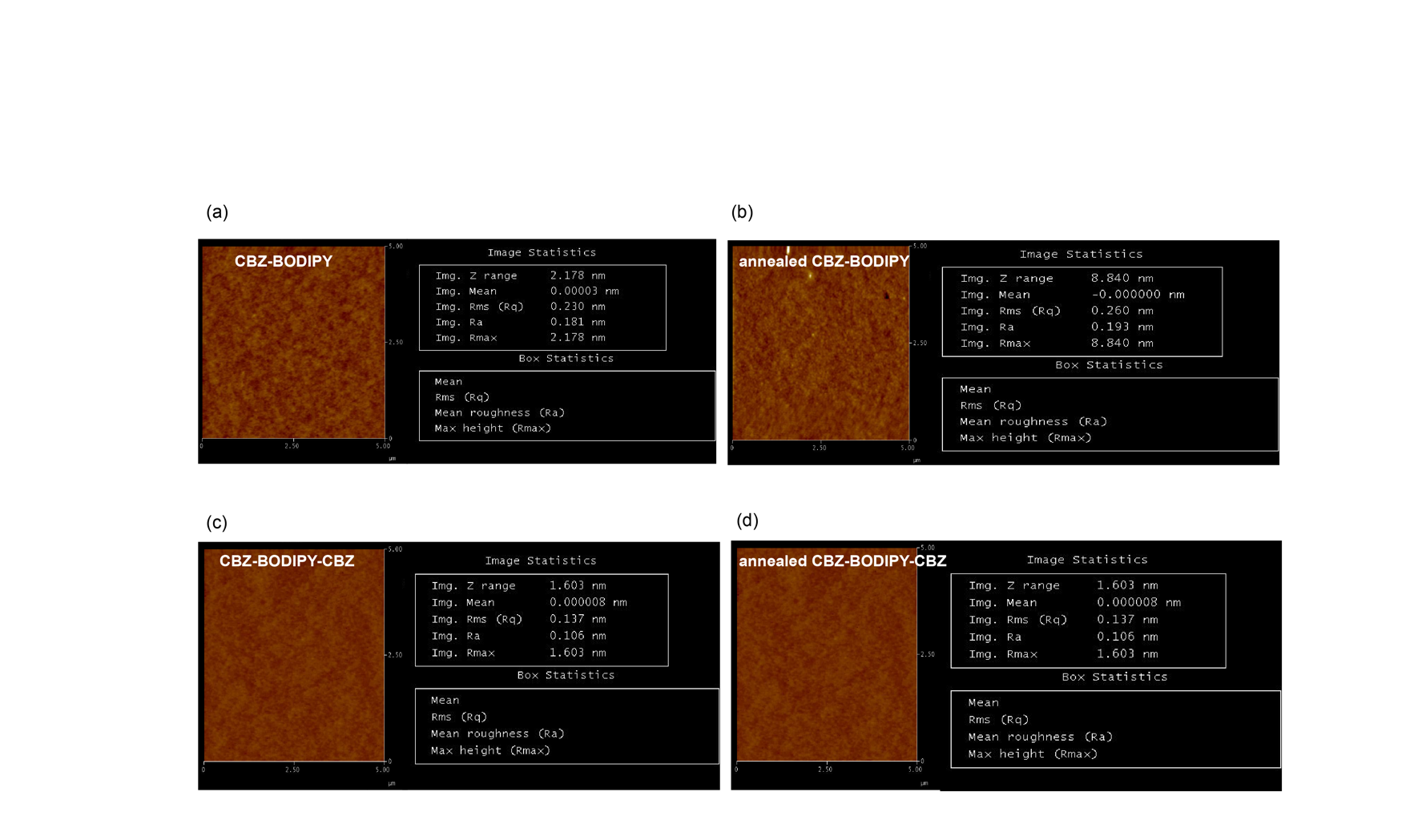


Surface roughness CBZ-BODIPY
